# GeSe ovonic threshold switch: the impact of functional layer thickness and device size

**DOI:** 10.1038/s41598-024-57029-7

**Published:** 2024-03-20

**Authors:** Jiayi Zhao, Zihao Zhao, Zhitang Song, Min Zhu

**Affiliations:** 1 State Key Laboratory of Functional Materials for Informatics, Shanghai Institute of Micro-System and Information Technology, Chinese Academy of Sciences, Shanghai, 200050 China; 2 University of Chinese Academy of Sciences, Beijing, 100029 China

**Keywords:** Ovonic threshold switch, GeSe, Scalability, Selector, Threshold switching field, Phase change memory, Nanoscale devices, Techniques and instrumentation

## Abstract

Three-dimensional phase change memory (3D PCM), possessing fast-speed, high-density and nonvolatility, has been successfully commercialized as storage class memory. A complete PCM device is composed of a memory cell and an associated ovonic threshold switch (OTS) device, which effectively resolves the leakage current issue in the crossbar array. The OTS materials are chalcogenide glasses consisting of chalcogens such as Te, Se and S as central elements, represented by GeTe_6_, GeSe and GeS. Among them, GeSe-based OTS materials are widely utilized in commercial 3D PCM, their scalability, however, has not been thoroughly investigated. Here, we explore the miniaturization of GeSe OTS selector, including functional layer thickness scalability and device size scalability. The threshold switching voltage of the GeSe OTS device almost lineally decreases with the thinning of the thickness, whereas it hardly changes with the device size. This indicates that the threshold switching behavior is triggered by the electric field, and the threshold switching field of the GeSe OTS selector is approximately 105 V/μm, regardless of the change in film thickness or device size. Systematically analyzing the threshold switching field of Ge–S and Ge–Te OTSs, we find that the threshold switching field of the OTS device is larger than 75 V/μm, significantly higher than PCM devices (8.1–56 V/μm), such as traditional Ge_2_Sb_2_Te_5_, Ag–In–Sb–Te, etc. Moreover, the required electric field is highly correlated with the optical bandgap. Our findings not only serve to optimize GeSe-based OTS device, but also may pave the approach for exploring OTS materials in chalcogenide alloys.

## Introduction

With the development of information technology in the era of big data, storage devices play an increasingly important role. Currently, flash memory and dynamic random access memory (DRAM) dominate the semiconductor memory market, accounting for more than 97% of the market^1,2^. However, high-density flash memory suffers from slow operation speed of approximately100 μs and poor endurance of around 10^4^ cycles, while ultrafast DRAM consumes too much energy during each 65 ms refresh process^3^. To overcome these defects, new memory technologies have been developed, such as resistive random access memory (RRAM)^4,5^, phase change memory (PCM)^6,7^, magnetic random access memory (MRAM)^8,9^ and ferroelectric random access memory (FRAM)^10,11^. Among these, PCM with a capacity of 128–256 Gb has been commercialized given its high density, fast speed and nonvolatile nature^12,13^.

3D X-Point architecture, implemented in commercial 3D PCM, has been verified to be the best solution to achieve high-density storage, in which the key is the utilization of a two-terminal selector device known as ovonic threshold switch (OTS)^14,15^. The OTS exhibits excellent selective performance, and its fabrication process is highly compatible with PCM. The requirements for OTS are strict, such as ≥ 10 mA/cm^2^ working current, > 10^4^ selectivity (On-current/Off-current), as well as nanosecond switching speed^16–18^. The OTS device has a sandwich structure, in which the middle layer is chalcogenide glass, called OTS material. Although hundreds of OTS materials have been found since first discovery in 1964^19^, the OTS materials cannot function without Te, Se and S chalcogens^17^, represented by GeTe_6_^18,20^, GeSe^21–23^ and GeS^24,25^, respectively. Among them, As and Si co-doped GeSe OTS materials are believed to be the ones successfully utilized in commercial 3D PCM^26,27^. However, the scalability of GeSe OTS still has not been thoroughly investigated, which inhibits the enhancement of storage capacity through device miniaturization^28–30^. Therefore, in this work, the scalability characteristics in functional layer thickness and device size^31–34^ of traditional GeSe devices was investigated, which provides solutions for optimizing device’s performance. Subsequently, the relationship between threshold switching field and optical bandgap was discovered, providing an effectively scheme for distinguishing OTS and PCM materials.

## Experiment

We prepared GeSe T-shaped devices with different functional layer thicknesses and TiN-bottom electrode diameters. All the transition films were deposited by sputtering of GeSe target at room temperature, while the sputtering power of target was 20 W. The sputtering speed was measured through dividing thickness by time using scanning electron microscopy (SEM), which was ~ 1.45 nm/min. Besides, bottom TiN electrodes with different sizes were fabricated by 45 nm complementary metal oxide semiconductor (CMOS) technology. Finally, TiN was sputtered as top electrode. There were mainly two instruments used in studying the devices, which were Keithley 4200A-SCS parameter analyzer and MSO54 mixed signal oscilloscope. The former was for electrical performance, while the latter was used to capture the input pulses and device responses. The lamella used for transmission electron microscopy (TEM) investigation was prepared by focused ion beam (Helios, G4), which then was investigated by Cs-corrected TEM (JEOL, ARM 300).

## Results and discussion

**Figure 1:**
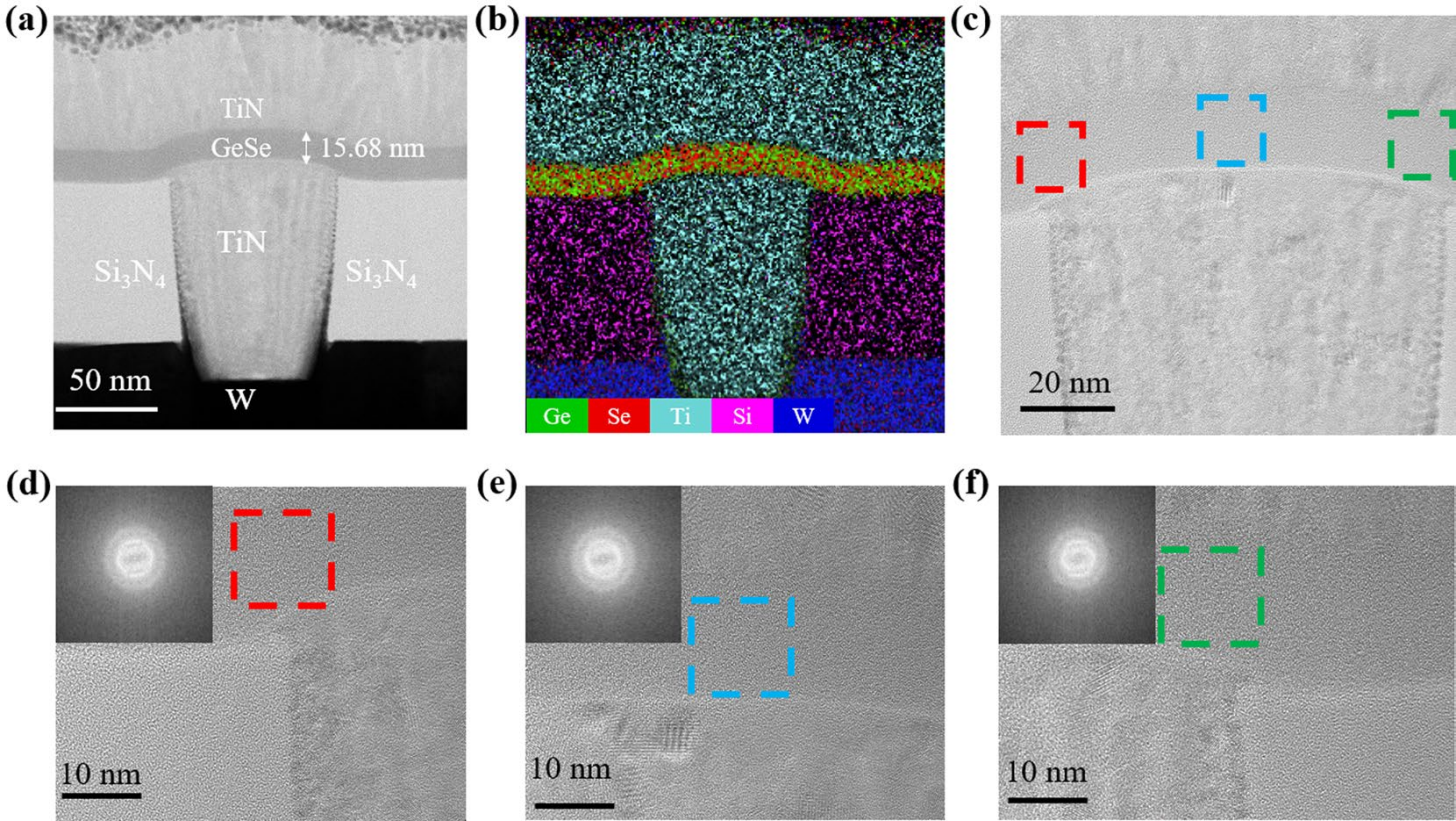
(**a**) Cross-sectional TEM of a GeSe device with a ~ 60 nm TiN plug. (**b**) EDS mapping of Ge, Se, Ti, N, Si and W. (**c**) Three different places of GeSe layer for local amplification. (**d–f**) HAADF-STEM image of GeSe layer at three locations, inset is the FFT image.

Figure 1a shows the cross-sectional TEM image of the T-shaped device with a ~ 15 nm-thick GeSe film^35^. The ratio of Ge and Se elements in the film was approximately 54:46, which is close to 1:1. Next, the energy-dispersive spectroscopy (EDS) mappings were performed, including Ge, Se, Ti, Si and W elements, as shown in Fig. 1b. Different elements are homogeneously distributed without segregation or diffusion. To further investigate whether the GeSe film crystallized during fabrication process, the high angle annular dark filed-scanning transmission electron microscopy (HAADF-STEM) images of GeSe film in three areas were observed. Figure 1d–f are local magnification of the corresponding positions in Fig. 1c in turn. The inset demonstrates fast Fourier transform (FFT) image. No obvious halo is observed around the bright spots in the center, indicating that the GeSe film is in an amorphous state.

**Figure 2:**
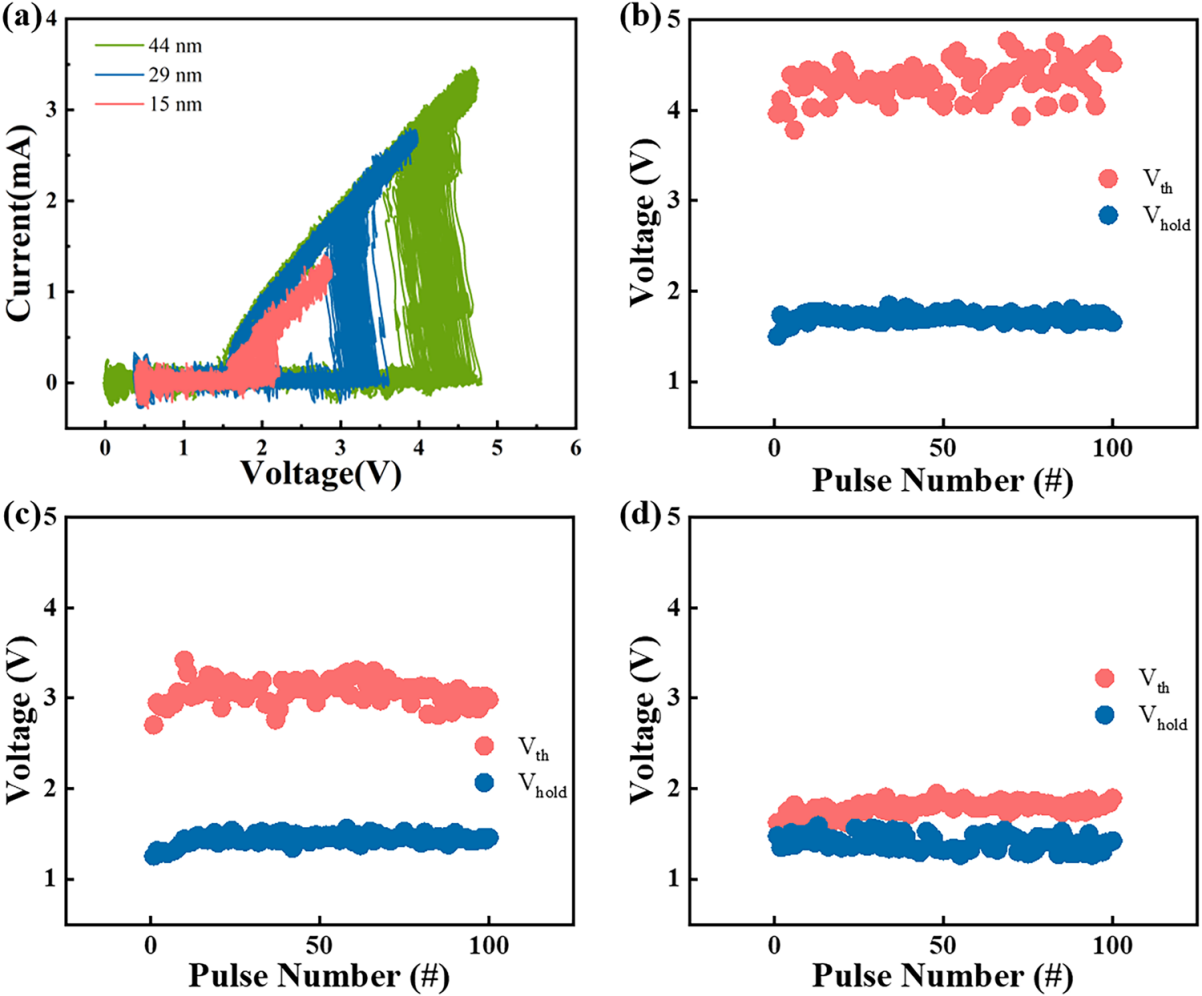
(**a**) *I–V* curves of different GeSe devices subjected to 100 consecutive triangular pulses after fire process. (**b–d**) Distribution of *V*_th_ and *V*_hold_ with different GeSe layer thicknesses of 44, 29 and 15 nm, respectively.

Firstly, the scalability of functional layer thickness is studied through a series of electrical tests. Then, the current–voltage (*I–V*) behaviors of the GeSe device with all three functional layer thicknesses under 100 continuous triangular pulses were performed, as displayed in Fig. 2a. The electrode of all devices tested in this part is 200 nm. Taking 44 nm-thick GeSe device for instance, it suddenly transits to low resistance state between 4–5 V, which is called the threshold voltage (*V*_th_). The fluctuation range of V_th_ is within 1 V, which is relatively small, demonstrating the device is stable and the results concerning scalability are reliable. After that, as the voltage decreases to 1–2 V, the device returns to the high resistance state, which is called the holding voltage (*V*_hold_). Before normal switching, the device needs to be initialized by a stronger amplitude of voltage, which is called the fire voltage (V_fire_)^36,37^. During the fire process, traps in the bandgap are rearranged, and thus the local state density change, which require more energy than normal switching circle^38^. Figure 2b–d show the distribution of *V*_th_ and *V*_hold_. *V*_th_ of devices with thickness of 44, 29 and 15 nm is roughly maintained at 3.8–4.8 V, 2.7–3.5 V and 1.5–2 V, respectively. Obviously, the *V*_th_ of the device almost lineally decreases as the GeSe layer thickness decreases. Meanwhile, the *V*_hold_ of the device with three different GeSe thicknesses is distributed between 1.1 and 1.7 V with minor decline.

**Figure 3:**
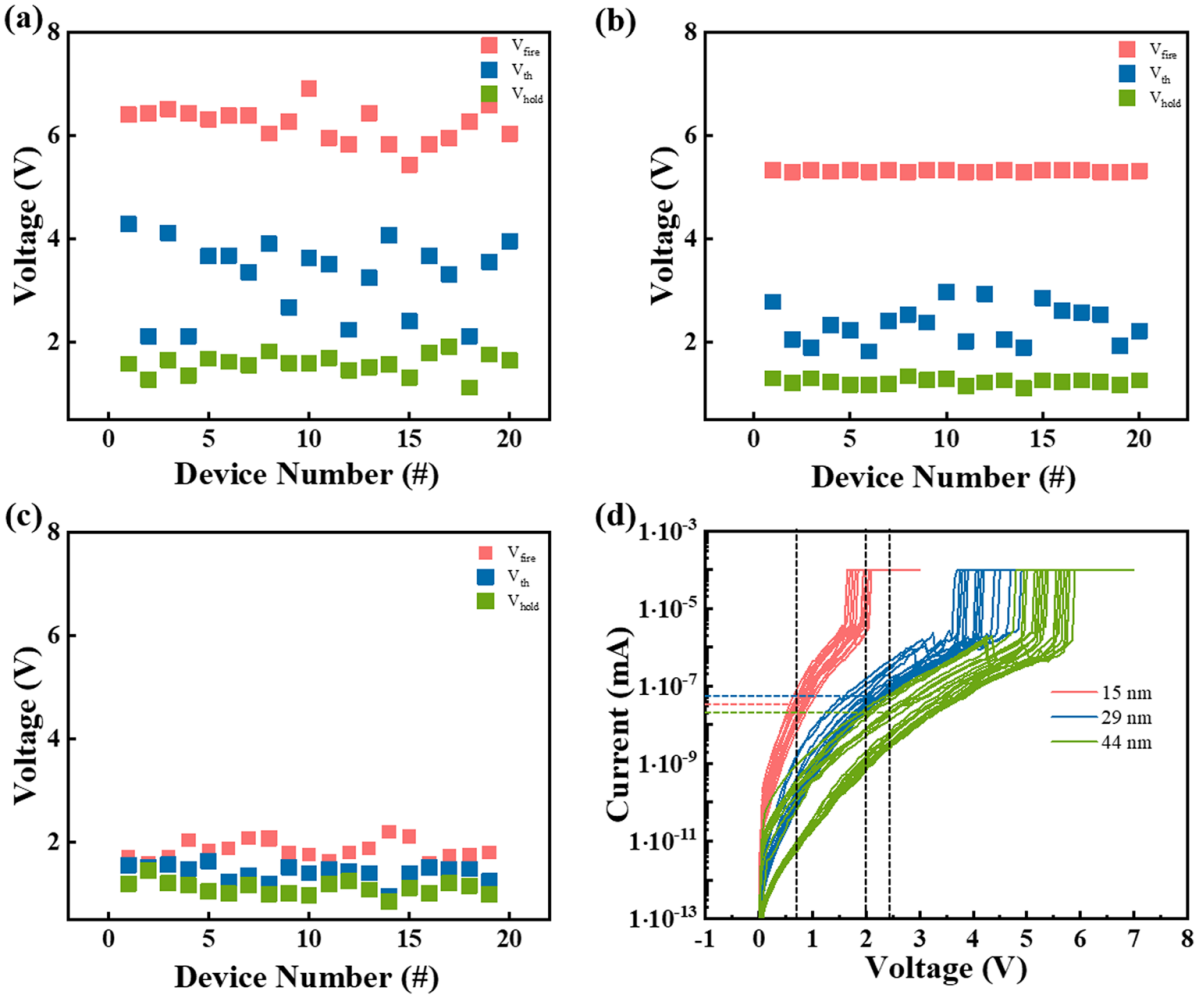
(**a–c**) Distribution of *V*_fire_, *V*_th_ and *V*_hold_ with different GeSe layer thicknesses of 44, 29 and 15 nm, respectively. (**d**) DC *I–V* curves of GeSe devices in series with all three thicknesses.

It is also necessary to carry out experiments with different devices to increase the universality of the conclusion. As shown in Fig. 3a–c, four triangular pulses were applied to 20 different devices, including one fire pulse with higher voltage amplitude and three threshold pulses with smaller amplitude. The fire voltage of the device under the first pulse, as well as the threshold voltage and holding voltage of the device under the last pulse are counted. It could be seen that *V*_fire_ of 44, 29 and 15 nm thickness devices is distributed in 5.5–7 V, 5–5.5 V and 1.7–2.2 V with minor fluctuation. Combined with the previous conclusion, the *V*_fire_ and *V*_th_ of the device gradually increases with the increase of the thickness. Afterwards, Fig. 3d displays direct current (DC) *I-V* tests of GeSe device with different thicknesses and the leakage current at 1/2*V*_th_ is calculated. The leakage current, also known as *I*_off_, plays a critical role in the storage array, which directly determines the power consumption of the whole chip and the integration density of the array^18,39–41^. *I*_off_ is 2.4 × 10^–8^, 4.94 × 10^–8^ and 2.54 × 10^–8^ A of 44, 29 and 15 nm thickness device, respectively. It’s easy to observe the variation of switching voltage with different thicknesses, but the leakage current shows no obvious change.

**Figure 4:**
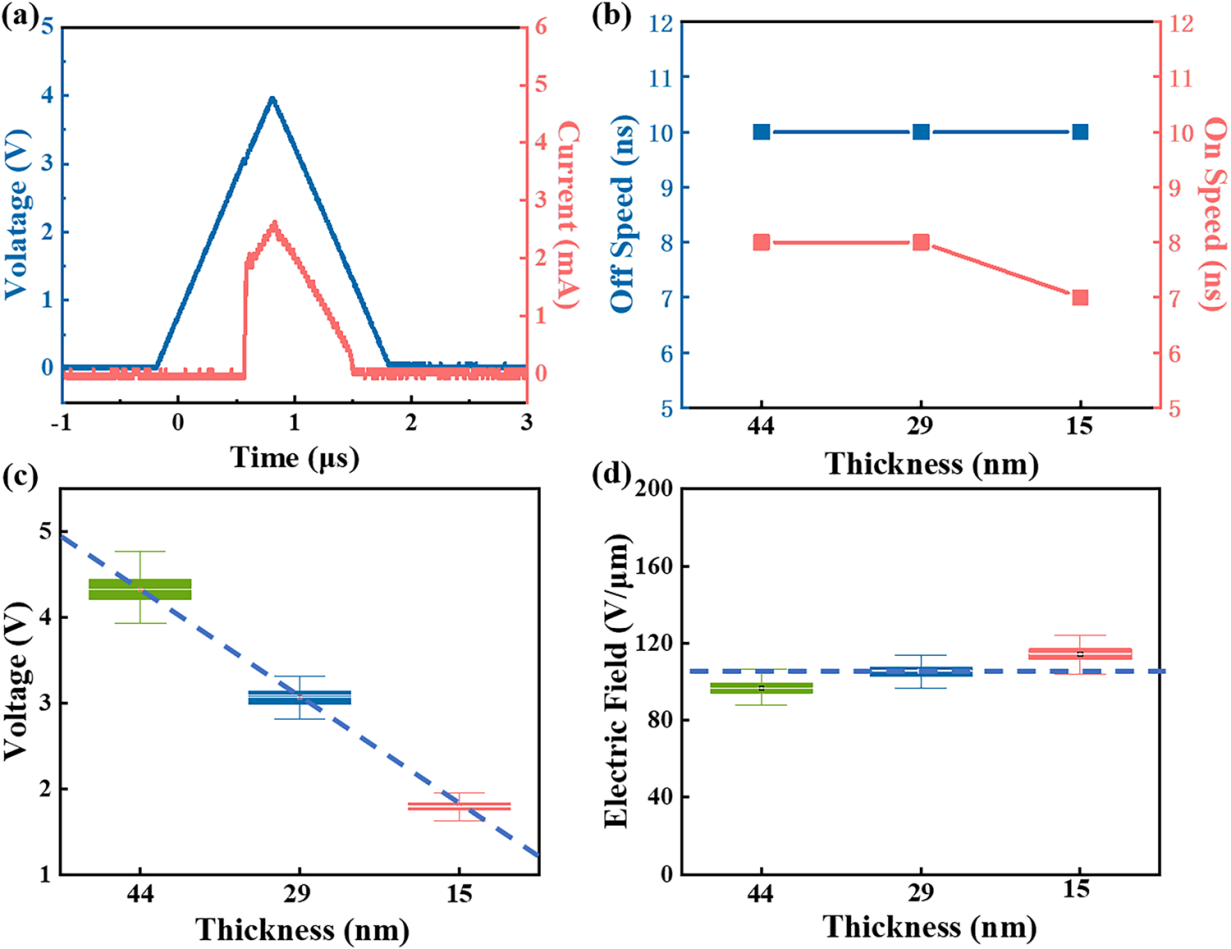
(**a**) Triangular pulse and corresponding curve when measuring device’s speed. (**b**) Statistical switching speeds distribution of various GeSe devices. (**c**) Relationship between threshold voltage of GeSe device and thickness of transition layer. (**d**) Relationship between threshold switching field of GeSe device and transition layer thickness.

As shown in Fig. 4a, the oscilloscope is used to capture the instantaneous responses of the device, enabling the measurement of the device’s on and off-speed. An abrupt rise of current demonstrates the device is turned on and transits from a high resistance state to a low resistance state, while the time span represents the on-speed. Similarly, the time span of a sudden drop in current refers to the off-speed. In Fig. 4b, the on-speed of 44, 29 and 15 nm-thick devices are 8, 8 and 7 ns, while the off-speed of all three devices is 10 ns. To explore the transition mechanism of GeSe OTS selector, the threshold voltages and threshold switching field of different devices are calculated, where the electric field is obtained through dividing the threshold voltages calculated with 100 continuous pulses by the actual thickness of the film. Figure 4c illustrates the threshold voltage decreases linearly with the decrease of the GeSe layer thickness. In addition, the threshold switching field shows no significant change with the decrease of film thickness, maintaining a value around 105 V/μm as shown in Fig. 4d.

**Figure 5:**
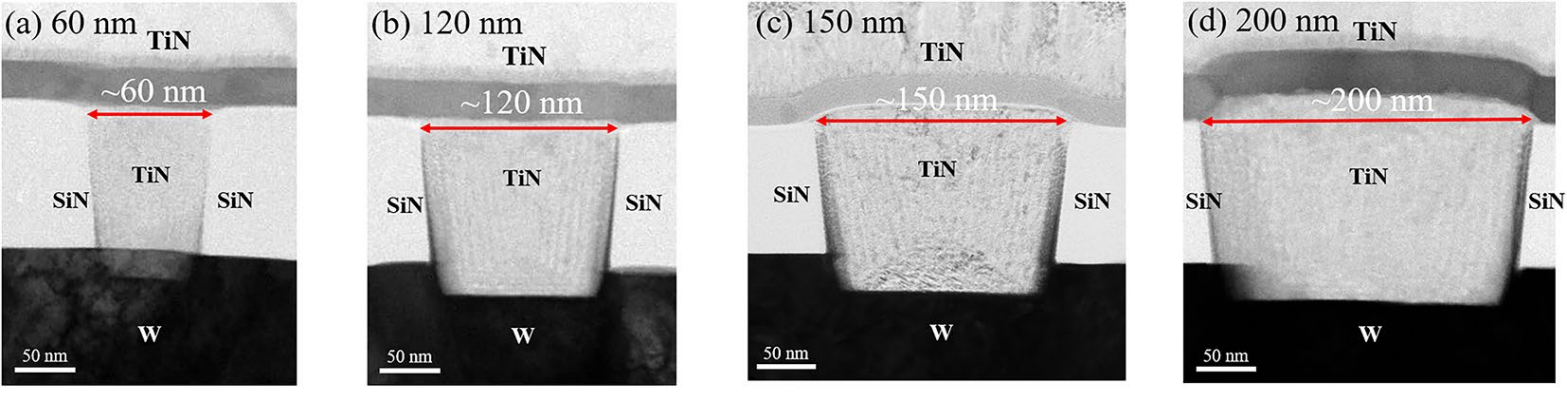
Cross-sectional TEM images of GeSe devices with ~ 60, 120, 150 and 200 nm TiN plug, respectively.

Secondly, with the extension of Moore’s law, the device gradually shrinks. As the device is three-dimensional, it’s necessary to investigate its scalability characteristics in terms of both thickness and size. Therefore, exploring the scalability of the OTS device sizes is of great significance for the integration of 3D high-density storage arrays^42,43^. In our work, as shown in Fig. 5, the GeSe layers are deposited on TiN bottom electrodes with diameters are 60, 120, 150 and 200 nm, respectively. Then, TiN is sputtered as the top electrode, at the bottom is the W interconnects, and TiN/GeSe/TiN layers are among them. An isolation layer is on both sides of the bottom electrode to isolate each cell.

**Figure 6:**
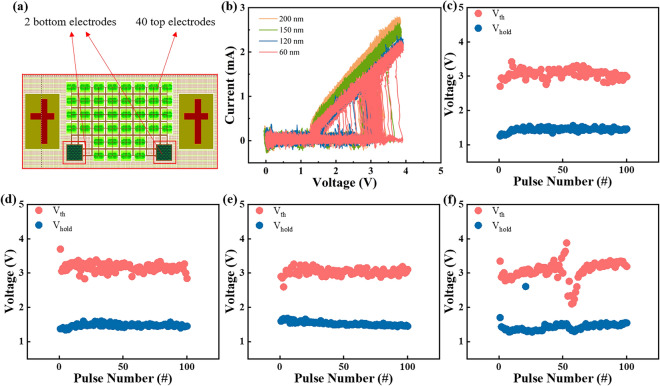
(**a**) Structure of arrays in the chip. (**b**) *I–V* curves of different GeSe devices subjected to 100 consecutive triangular pulses after fire process. (**c–f**) Distribution of *V*_th_ and *V*_hold_ with different electrode sizes of 200, 150, 120 and 60 nm, respectively.

The structure of arrays in the chip is composed of 2 bottom electrodes and 40 top electrodes, besides the cross structure on the left and right sides is for better alignment during lithography as shown in Fig. 6a. For electrical tests using Keithley 4200A-SCS parameter analyzer, two probes were pressed against the larger bottom electrode and the smaller top electrode. Triangular pulses were applied to devices with varying electrode sizes. From Fig. 6b, it is evident that *V*_th_ of devices with electrode sizes of 200, 150, 120 and 60 nm is distributed in 2–4 V. Moreover, the *V*_th_ does not vary significantly with changes in electrode size, indicating that *V*_th_ remains stable. Figure 6c–f display the specific distribution of *V*_th_ and *V*_hold_, which is relatively concentrated and basically unchanged, demonstrating the high stability of the device. Meanwhile, the device with 60 nm electrode size shows wide discrete range of *V*_th_, indicating the stability is worse than that of other devices with different electrode sizes, which needs to be improved by optimizing the manufacturing process.

**Figure 7:**
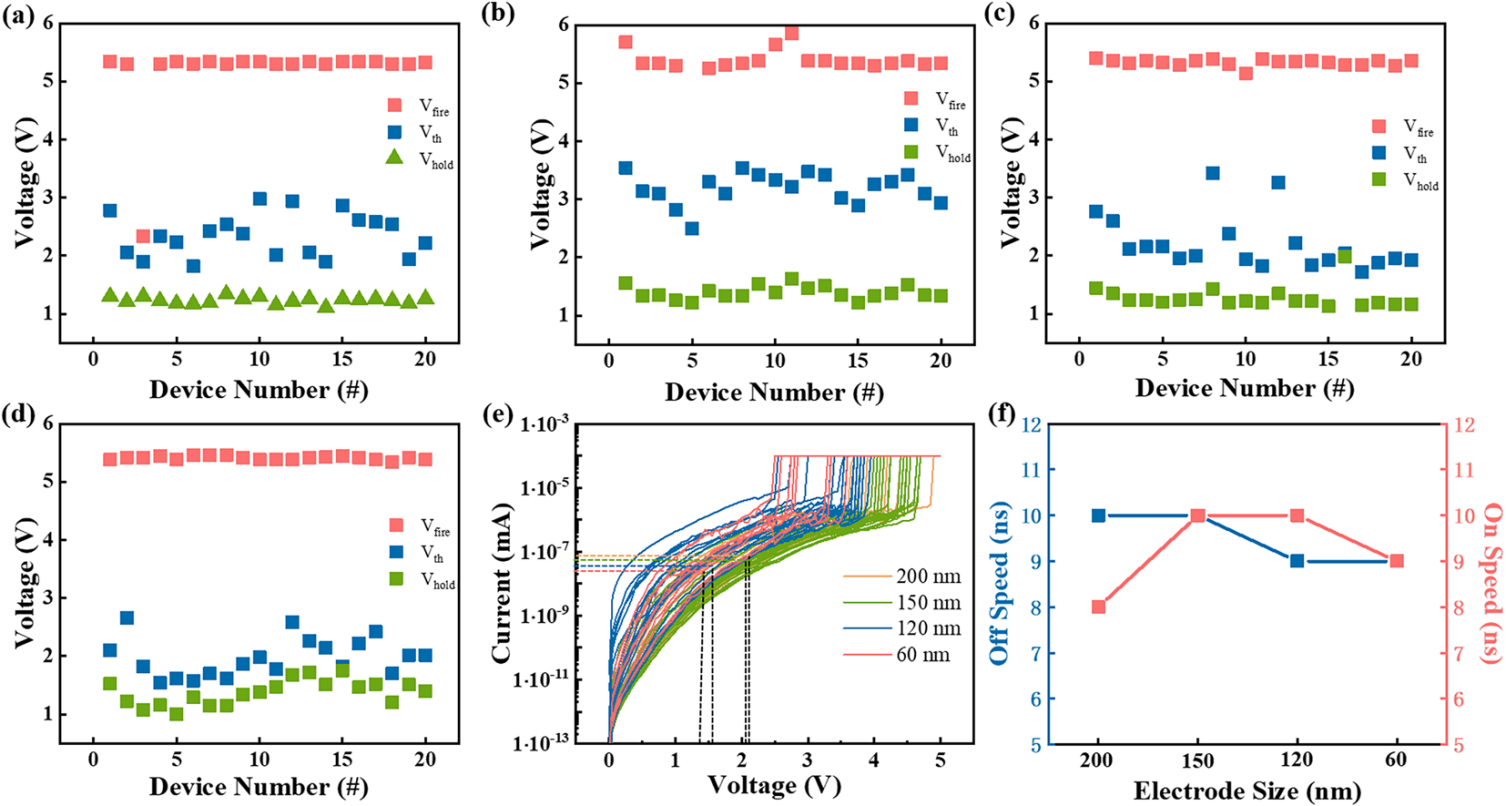
(**a–d**) Distribution of *V*_fire_, *V*_th_ and *V*_hold_ with different electrode sizes of 200, 150, 120 and 60 nm, respectively. (**e**) DC *I-V* curves of GeSe devices in series with all four electrode sizes. (**f**) Statistical switching speeds distribution of various GeSe devices.

Electrical tests were performed on 20 different devices at each electrode size, and the corresponding *V*_fire_, *V*_th_ and *V*_hold_ were recorded, as shown in Fig. 7a–d. *V*_fire_, *V*_th_ and *V*_hold_ of the four devices is 5–6 V, 1.5–3.5 V and 1–2 V with small fluctuations, showing the voltage distribution of the four devices is similar to each other. Figure 7e shows the DC* I–V* tests. *I*_off_ of the device with electrode size of 200, 150, 120 and 60 nm is 5.65 × 10^–8^ A, 4.59 × 10^–8^ A, 3.265 × 10^–8^ and 2.35 × 10^–8^ A. That is, as the device size decreases, the leakage current also diminishes. Therefore, reducing the electrode size of the device is a helpful way to improve the storage density of the array. At last, the switching speed is measured, and Fig. 7f displays the comparison of various devices. The on-speed of GeSe device with electrode size of 200, 150, 120 and 60 nm is 8, 10, 10 and 9 ns, while the off-speed is 10, 10, 9 and 9 ns, respectively. Compared with Fig. 4b, both the on-speed and off-speed are distributed between 7 and 10 ns for all devices. Not only is the speed of the device measured manually with an error range of 1–2 ns, but there is also no order of magnitude difference. Therefore, the subtle difference could be ignored, and the switching speed is not related to the device variability.

**Figure 8:**
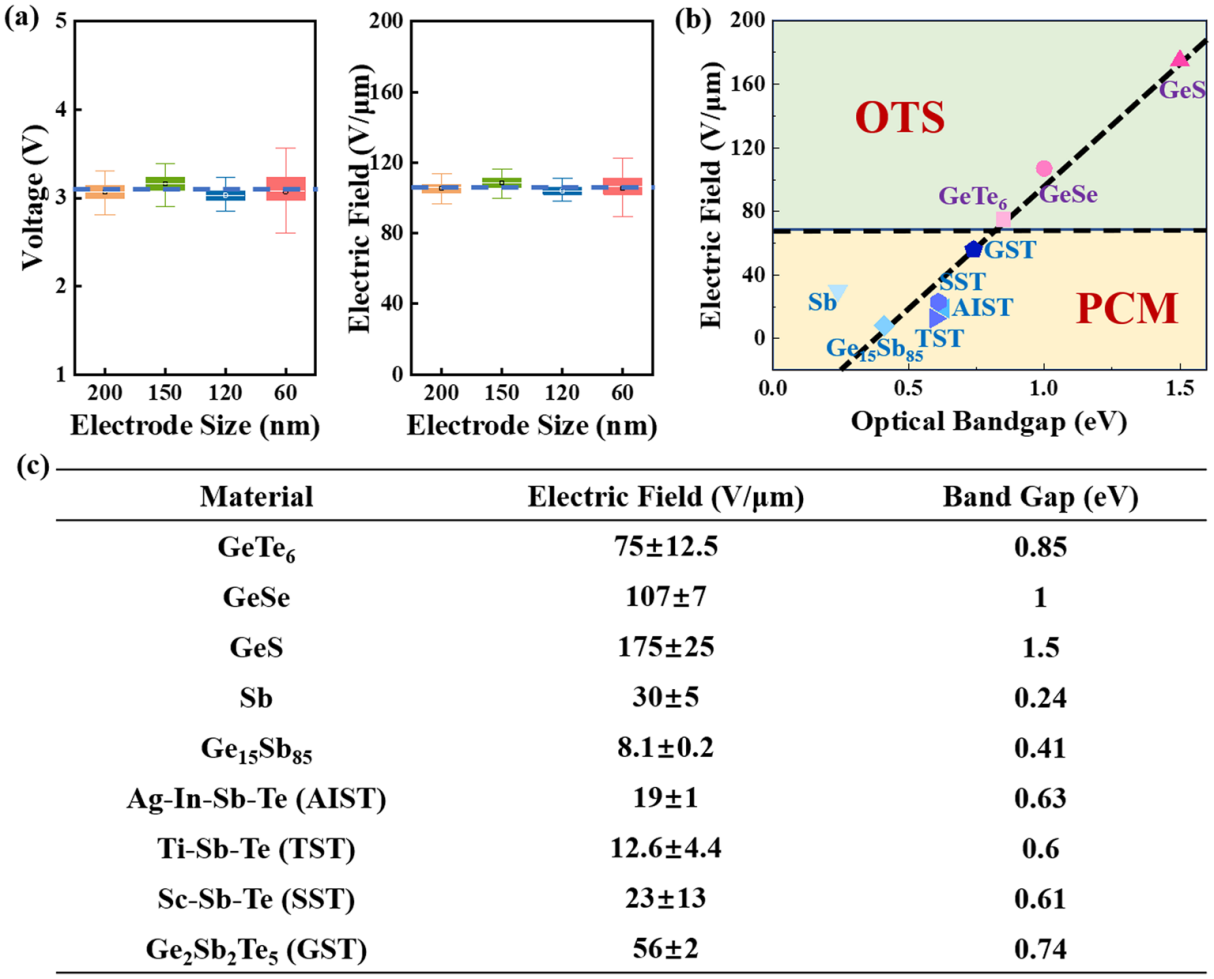
(**a**) The tendency of GeSe device’s threshold voltage and electric field with electrode size changing. (**b**) Intuitive comparison on the relationship between electric field and optical bandgap of common OTS and PCM materials. (**c**) Electric field and optical bandgap values of common OTS and PCM materials.

Figure 8a displays the *V*_th_ and threshold switching field of GeSe devices with different electrode sizes. Obviously, although the device size changes, *V*_th_ and electric field distribution are basically along a horizontal line. Specifically, *V*_th_ and the threshold switching field are distributed at around 3 V and 105 V/μm, respectively. Typically, *V*_th_ is used to describe the critical point at which OTS and PCM change from high resistance to low resistance. However, due to the different thicknesses of functional layer, *V*_th_ can change greatly. Therefore, the average threshold switching field of common OTS and PCM devices is statistically analyzed, effectively avoiding the influence of the functional layer’s thickness, as shown in Fig. 8b. Moreover, in Fig. 8c, the detail data of the materials investigated is listed. Three OTS materials, GeTe_6_, GeSe and GeS, exhibit electric field ranging from 75 to 175 V/μm^24^. In addition, the average threshold switching field of six PCM materials, Ge_15_Sb_85_, Ti–Sb–Te (TST), Ag–In–Sb–Te (AIST), Sc–Sb–Te (SST), Sb and Ge_2_Sb_2_Te_5_ (GST), is distributed in 8.1–56 V/μm^33,44–48^. Threshold voltage increases with the broaden of bandgap, regardless of traps^25^. Thus, for different kinds of devices with same thickness, the one with wider bandgap exhibits larger threshold transition field. Noticeably, the electric field of OTS is above 70 V/μm, significantly larger than that of PCM (< 60 V/μm). However, regardless of OTS or PCM, the electric field is approximately linear with the optical bandgap. Besides, the OTS materials typically possess a bandgap greater than that of PCM materials. This characteristic may be utilized as a criterion for screening potential OTS materials candidates in chalcogenides.

## Conclusions

In this paper, the scalability characteristics of traditional GeSe OTS selector are comprehensively investigated, including functional layer thickness scalability and electrode size scalability. On one hand, when the thickness of the functional layer is reduced from 44 to 15 nm, *V*_fire_, *V*_th_ and *V*_hold_ decreases from 7 to 2.2 V, 4.8 to 2 V and 1.8 V to 1.2 V, respectively. Besides, *I*_off_ is basically maintained at 30 nA and the electric field remained around 105 V/μm, both of which don’t change with the variation of the thickness. On the other hand, as the device size decreases from 200 to 60 nm, *V*_fire_, *V*_th_, *V*_hold_ and electric field is distributed at 5–6 V, 1.5–3.5 V, 1–2 V and around 105 V/μm, remaining unchanged on the whole. At the same time, *I*_off_ decreases from 56.5 to 23.5 nA. In summary, the electric field of GeSe OTS selector is basically unchanged regardless of thickness or size reduction, indicating an electric field-driven switching mechanism. Meanwhile, the leakage current decreases with the reduction of the device size, and the switching voltage decreases with the reduction of the thickness, reflecting its great potential in large-scale high-density memory. To sum up, the 15 nm thick device with the electrode size of 60 nm demonstrates the best performance. Finally, it is concluded that the threshold switching field increases linearly with the increase of optical bandgap. The OTS materials typically possess a bandgap larger than PCM materials. This characteristic may be utilized as a criterion for screening potential OTS material candidates in chalcogenides.

## Data Availability

The data that support the findings of this study are available from the corresponding author upon reasonable request.
